# Prediction of high-risk emergency department revisits from a machine-learning algorithm: a proof-of-concept study

**DOI:** 10.1136/bmjhci-2023-100859

**Published:** 2024-04-22

**Authors:** Chih-Wei Sung, Joshua Ho, Cheng-Yi Fan, Ching-Yu Chen, Chi-Hsin Chen, Shao-Yung Lin, Jia-How Chang, Jiun-Wei Chen, Edward Pei-Chuan Huang

**Affiliations:** 1 Department of Emergency Medicine, National Taiwan University Hospital Hsin-Chu Branch, Hsinchu, Taiwan; 2 Department of Emergency Medicine, College of Medicine, National Taiwan University, Taipei, Taiwan; 3 Institute of Information Science, Academia Sinica, Taipei, Taiwan; 4 Institute of Information Systems and Applications, National Tsing Hua University, Hsinchu, Taiwan; 5 Department of Emergency Medicine, National Taiwan University Hospital Yun-Lin Branch, Douliou, Taiwan; 6 Department of Emergency Medicine, National Taiwan University Hospital, Taipei, Taiwan

**Keywords:** Machine Learning, Patient Outcome Assessment

## Abstract

**Background:**

High-risk emergency department (ED) revisit is considered an important quality indicator that may reflect an increase in complications and medical burden. However, because of its multidimensional and highly complex nature, this factor has not been comprehensively investigated. This study aimed to predict high-risk ED revisit with a machine-learning (ML) approach.

**Methods:**

This 3-year retrospective cohort study assessed adult patients between January 2019 and December 2021 from National Taiwan University Hospital Hsin-Chu Branch with high-risk ED revisit, defined as hospital or intensive care unit admission after ED return within 72 hours. A total of 150 features were preliminarily screened, and 79 were used in the prediction model. Deep learning, random forest, extreme gradient boosting (XGBoost) and stacked ensemble algorithm were used. The stacked ensemble model combined multiple ML models and performed model stacking as a meta-level algorithm. Confusion matrix, accuracy, sensitivity, specificity and area under the receiver operating characteristic curve (AUROC) were used to evaluate performance.

**Results:**

Analysis was performed for 6282 eligible adult patients: 5025 (80.0%) in the training set and 1257 (20.0%) in the testing set. High-risk ED revisit occurred for 971 (19.3%) of training set patients vs 252 (20.1%) in the testing set. Leading predictors of high-risk ED revisit were age, systolic blood pressure and heart rate. The stacked ensemble model showed more favourable prediction performance (AUROC 0.82) than the other models: deep learning (0.69), random forest (0.78) and XGBoost (0.79). Also, the stacked ensemble model achieved favourable accuracy and specificity.

**Conclusion:**

The stacked ensemble algorithm exhibited better prediction performance in which the predictions were generated from different ML algorithms to optimally maximise the final set of results. Patients with older age and abnormal systolic blood pressure and heart rate at the index ED visit were vulnerable to high-risk ED revisit. Further studies should be conducted to externally validate the model.

**WHAT IS ALREADY KNOWN ON THIS TOPIC**

- High-risk emergency department (ED) revisits can potentially be prevented in advance. However, the predictive model for high-risk ED return is yet to be determined in this particular cohort.

**WHAT THIS STUDY ADDS**

- Four machine-learning models were employed to predict high-risk ED revisits. Among these, the stacked ensemble algorithm demonstrated superior predictive performance compared with the other artificial intelligence (AI) models, achieving an area under the receiver operating characteristic curve value of 0.82. Notably, all AI models outperformed the traditional logistic regression model in terms of predictive accuracy.

**HOW THIS STUDY MIGHT AFFECT RESEARCH, PRACTICE OR POLICY**

- ED physicians should identify patients potentially at high risk for ED revisits to prevent further deterioration of their condition.

## Introduction

Emergency department (ED) revisit is a well-known quality index for ED medical care and patient safety, in which revisit rates >5% may reflect poor quality of care, and those <1% indicate undue risk aversion.^1^ Previous studies indicated that ED revisit may increase medical costs, ED crowding and poor prognosis, particularly in patients who require hospital admission, often due to rapid deterioration after ED discharge.^2–4^ Over the past decade, this concept has become challenging because the factors that influence ED revisit are multifactorial, such as issues related to diagnosis, management, procedural complications and medical adverse effects.^3 5 6^ Most issues are preventable and do not result in severe outcomes.^7^ Recent studies of intrinsic factors for high-risk ED revisit focused on patients who received hospital admission or intensive care unit (ICU) care.^8–10^

To identify potential risk factors for either high-risk ED revisit or unscheduled ED revisit within 72 hours, logistic linear regression models are widely used. Well-known factors for high-risk ED revisit include age, male sex, ambulance transport for return visit, longer ED length of stay, symptoms of dyspnoea or chest pain on ED presentation, triage level 1 or 2, acute change in levels of consciousness and unstable vital signs (tachycardia and/or fever), among others.^8 11 12^ However, a study of ED revisit has been limited by the use of linear algorithms, such as logistic regression routine, use of administrative data and small sample sizes, in part because the assessment of risk factors is more complicated than that possible with linear association.^13^

With the development of artificial intelligence, the machine-learning (ML)-based prediction model is used now as a clinical classifier. Lee *et al* developed an ML framework combining a particle swarm optimisation feature selection algorithm and an optimisation-based discriminant analysis model, to predict ED revisit. Hong *et al* indicated that gradient-boosting models that leveraged clinical data were superior to traditional logistic regression models built on administrative data to predict ED revisit.^14^ Hsu *et al* developed an ML model, the voting classifier model, to predict ED revisit in patients with abdominal pain.^15^ These works shed light on the use of a prediction model for ED revisit based on an ML algorithm.

In previous ML-based studies, the work by Lee and Hong focused on building a prediction model for general ED revisit, whereas that of Hsu focused on ED revisit and abdominal pain symptoms. All prediction models showed superior prediction performance than that with a traditional logistic regression model. Expanding on these previous works, in the current study, we specifically predict high-risk ED revisit in 72 hours using a large dataset of adult ED revisits, with more than 150 variables extracted per visit from each medical record. Our study used a powerful classification algorithm—the stacked ensemble model. Also, a comprehensive comparison between models and previous reports is presented.

## Material and methods

### Study design, participants and setting

This study recruited patients who demonstrated unscheduled ED revisit within 72 hours between January 2019 and December 2021 from National Taiwan University Hospital Hsin-Chu Branch (NTUH-HCH), a tertiary centre with 829-bed capacity and more than 1700 staff. About 60 000 patients visit the ED each year; on average, 4.5% of these patients demonstrated an ED revisit after the index discharge. Patients were eligible for recruitment and analysis if they were age 20 years or older and demonstrated an ED revisit within 72 hours, whereas those who demonstrated ED revisit simply for diagnostic certificate or legal issue were immediately excluded.

### Data source, features and preprocessing

For data acquisition, independent ED attending physicians retrospectively reviewed the medical charts rather than extracting information from the integrated medical database to minimise the biases and errors in the original medical record. For data dimensions, 150 features were initially included, such as age, sex, pre-existing diseases, diagnosis, final disposition and two sets of covariates from the ED index and revisit. Each set contained triage level, vital signs, chief concern, management, medication and laboratory data. Pre-existing diseases were hypertension, diabetes mellitus, coronary artery disease, cerebrovascular disease, chronic kidney disease, malignancy, chronic obstructive pulmonary disease and previous documented surgery.

Triage level was determined by the Taiwan Triage and Acuity Scale computerised triage system, which has been validated with levels 1–5 to indicate resuscitation, emergent, urgent, less urgent and non-urgent.^16^ Vital signs included body temperature, respiratory rate, heart rate, blood pressure and oxygen saturation.

Chief concerns, originally written on medical charts, were recorded and classified by ED attending physicians into 30 common concerns, such as headache, vertigo, chest pain, short of breath, cough, rhinorrhoea, abdominal pain, nausea, vomiting, diarrhoea, dysuria, frequent urination, retention of urine, chills, limb oedema and tube malfunction, among others.

Management included electrocardiography, chest radiography, CT, MRI, panendoscopy, colonoscopy and specialist consultation with any formal consultation from surgeons, radiologists or intensivists. Medications included analgesics and antibiotics, either orally or intravenously. Laboratory data included serum concentrations of white cell count, haemoglobin, sodium, potassium and C reactive protein; blood gas analysis; and liver function and renal function tests. Diagnosis was categorised into infection, neurological diseases, circulation diseases, respiratory disease, gastrointestinal diseases, genitourinary diseases and musculoskeletal diseases. To predict high-risk ED revisit, the features in the index visit should be included, and those in the revisit should be reasonably excluded. A total of 79 features were used for data training (online supplemental figure 1).

10.1136/bmjhci-2023-100859.supp1 Supplementary data

For data cleaning, nonsense records were first removed. For unreasonable values for the feature, we re-examined the medical record to confirm the correctness. Because the rate of missing data was 4.3%, with most missing variables missing at random, mean imputation was used to replace missing values for a specific feature by the mean of non-missing cases for that feature. For data aggregation, we aggregated the feature according to its characteristic. We set body temperature as a binary feature based on whether it ranged between 36.0℃ and 37.4 ℃ or not. In addition, blood gas features (eg, pH value, partial pressure of carbon dioxide) were also aggregated for analysis.

### Stacked ensemble algorithm

**Figure 1:**
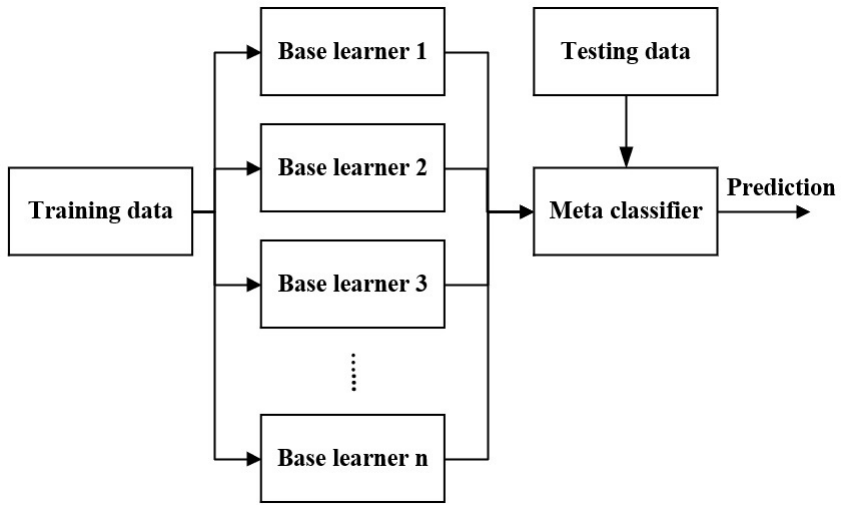
The stacked ensemble algorithm.

In ML, the ensemble method uses multiple learning algorithms, to achieve better predictive performance than that from any single-constituent learning algorithm.^17^ The principle of the ensemble method is to combine the predictions from multiple existing models or algorithms of the same or different types named after base learners, to further fine-tune the model. This approach creates a more robust system that combines the predictions from all base learners. By stacking multiple layers of ML models, each model carries its prediction to the layer above it, and the top layer model takes the final decision (figure 1).

### ML model and training

The ML model in this study included deep learning, random forest, extreme gradient boost (XGBoost) and stacked ensemble. The training set included 80% of subjects, while the remaining 20% of subjects were included in the testing set. To select the best model for the final testing dataset, we trained each model by 10-fold cross-validation. To increase the performance and prediction capacity according to our selected best base models, we proposed a stacked ensemble algorithm for the experiments in the base model. We performed hyperparameter tuning for each model. For deep learning, we used Bayesian optimisation based on the Gaussian process. For the random forest model, a random search algorithm was used because the decision tree was complex. For XGBoost, we tuned the hyperparameters based on Bayesian optimisation.

### Outcome measurement

For the ‘final disposition’ feature, high-risk ED revisit was defined as when a patient was admitted to hospital, including ICU admission or died, whereas low-risk ED revisit indicated a direct discharge after the return. Patients who were discharged against medical advice or transferred to other hospitals were excluded from the analysis.

### Statistical analysis

The features were computed by using SAS V.9.4 (SAS Institute). The Wilcoxon rank-sum test was applied to examine the significant differences among features when the features were continuous type, and the χ^2^ test was used for those that were categorical. A two-sided p<0.05 indicated statistical significance.

To compare performance between models, the area under the receiver operating characteristic curve (AUROC), accuracy, sensitivity and specificity were used. Accuracy indicated the number of high-risk and low-risk ED revisits that were correctly predicted. Sensitivity indicated the number of cases that were correctly predicted as high-risk ED revisit among all true high-risk ED revisits. Specificity indicated the number of cases that were correctly predicted as low-risk ED revisit among all low-risk ED revisits.

## Results

### Study flow and ML assignment

**Figure 2:**
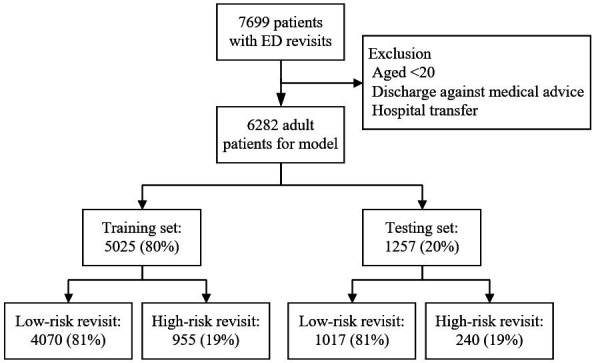
Flow chart for the inclusion of eligible subjects. ED, emergency department.

Figure 2 demonstrates the study population and assignment to the training and testing sets. A total of 7699 preliminary patients who demonstrated an unscheduled ED revisit within 72 hours were recorded. Patients aged younger than 20 years (n=1365, 17.7%) and those who demonstrated a discharge against medical advice (n=29, 0.4%) or hospital transfer (n=23, 0.3%) were excluded. After exclusion, 6282 adult patients were divided into two subgroups: 5025 for training (80.0%) and 1257 for testing (20.0%). High-risk ED revisit was found for 971 (19.3%) patients in the training set vs 252 (20.1%) in the testing set.

### Data from the training and testing cohort

Table 1 presents a comparison of characteristics between the training and testing cohorts. These cohorts were randomly selected from the population at an 80:20 ratio. In the training cohort, 971 patients (19.32%) experienced a high-risk revisit, compared with 252 patients (20.05%) in the testing cohort. There were no significant differences between the two cohorts in various aspects, including age, sex, year and month of enrolment, time from discharge to ED revisit, common pre-existing diseases, triage information, complaints and laboratory data. The process of randomisation achieved a balanced distribution across both cohorts.

**Table 1:** Comparison of demographics and medical information between training and testing cohorts

Variables	Training cohort (n=5025)	Testing cohort (n=1257)	P value
High-risk revisit	971 (19.32)	252 (20.05)	0.875
Age	58.6±19.6	58.1±19.8	0.382
Males	2650 (52.74)	664 (52.82)	0.923
Year			0.492
2019	1975 (39.30)	511 (40.65)	
2020	1547 (30.79)	389 (30.95)	
2021	1433 (28.52)	338 (26.89)	
Month			0.189
January	458 (9.11)	123 (9.79)	
February	409 (8.14)	102 (8.11)	
March	412 (8.20)	93 (7.40)	
April	374 (7.44)	107 (8.51)	
May	429 (8.54)	90 (7.16)	
June	384 (7.64)	105 (8.35)	
July	436 (8.68)	103 (8.19)	
August	433 (8.62)	136 (10.82)	
September	418 (8.32)	107 (8.51)	
October	449 (8.94)	92 (7.32)	
November	394 (7.84)	93 (7.40)	
December	359 (7.14)	87 (6.92)	
Return to ED			0.196
<24 hours	2357 (46.91)	554 (44.07)	
24–48 hour	1559 (31.02)	406 (32.30)	
48–72 hours	1039 (20.68)	278 (22.12)	
Pre-existing diseases			
Hypertension	1774 (35.30)	432 (34.37)	0.737
Diabetes mellitus	1067 (21.23)	268 (21.32)	0.931
Coronary artery disease	528 (10.51)	115 (9.15)	0.159
Cerebrovascular disease	221 (4.40)	45 (3.58)	0.201
Malignancy	802 (15.96)	213 (16.95)	0.386
Chronic kidney disease	374 (7.44)	86 (6.84)	0.471
COPD	153 (3.04)	50 (3.98)	0.093
Triage			
Glasgow Coma Scale (=15)	4783 (95.18)	1197 (95.23)	0.783
Triage level 1 or 2	807 (16.06)	195 (15.51)	0.647
Systolic blood pressure (mm Hg)	149.6±31.9	150.2±31.6	0.584
Diastolic blood pressure (mm Hg)	82.4±16.7	82.6±16.7	0.705
Pulse rate	91.6±19.6	92.1±20.0	0.493
Breath rate	20.2±2.8	20.3±2.8	0.791
Body temperature	36.9±0.8	36.9±0.8	0.362
Symptoms or complaints			
Headache	329 (6.55)	81 (6.44)	0.901
Chest pain	432 (8.60)	95 (7.56)	0.239
Dyspnoea	354 (7.04)	94 (7.48)	0.586
Abdominal pain	1097 (21.83)	253 (20.13)	0.194
Vomiting	503 (10.01)	116 (9.23)	0.412
Diarrhoea	253 (5.03)	66 (5.25)	0.748
Skin disorders	330 (6.57)	87 (6.92)	0.644
Leg oedema	128 (2.55)	28 (2.23)	0.518
Tube malfunction	241 (4.80)	65 (5.17)	0.575
Examination and blood data			
Electrocardiography	1554 (30.93)	415 (33.02)	0.144
Chest radiograph	2672 (53.17)	628 (49.96)	0.436
White cell count (x10ˆ9/L)	9.3±4.7	9.1±3.9	0.324
Neutrophil (%)	73.5±13.2	74.4±30.9	0.252
Haemoglobin (g/L)	128±26	129±25	0.192
Serum creatinine (mg/dL)	1.4±1.9	1.4±1.8	0.648

COPD, chronic obstructive pulmonary disease; ED, emergency department.

### Variable extraction and importance

The preliminary 151 features were included for screening, such as demographic data, pre-existing diseases and information on the index visit (month, visit time, triage level, vital signs and chief concerns) and the revisit (revisit time, triage level, vital signs, chief concerns, laboratory data and disposition). To provide a rationale and optimise the prediction for high-risk ED revisit, a total of 79 features, including the data from the index visit, were eventually included in the model for prediction.

**Figure 3:**
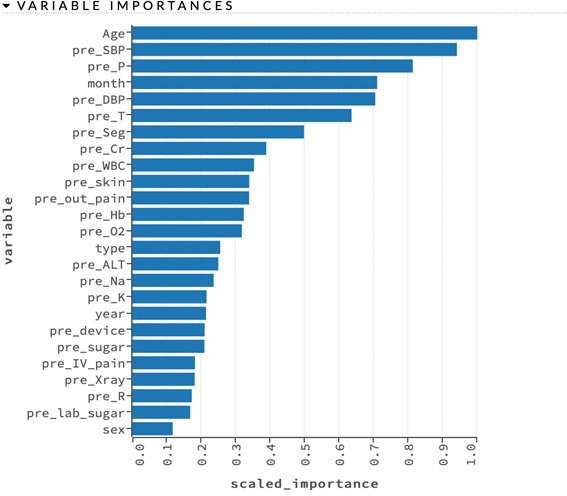
The scaled importance of the features.

Figure 3 shows the scaled importance of each variable. The importance variables were proposed based on the XGBoost model. Age was the most important feature for predicting high-risk ED revisit. In general, the leading features in the index visit other than age were systolic blood pressure, heart rate, month for visit, diastolic blood pressure and body temperature. For serum tests at the index visit, concentrations of neutrophils, creatinine and white cell count were important biomarkers to predict high-risk ED revisit, whereas alanine transaminase, sodium, potassium and glucose were minor ones. For chief concerns, only skin-related concerns or medical device issues contributed to high-risk ED revisit. For the physician’s management at the index visit, the feature of oral analgesic administration after discharge was a factor for high-risk ED revisit, but with low-scale importance. Patient sex was less relevant than other factors for high-risk ED revisit.

### Performance comparison of each model

**Figure 4:**
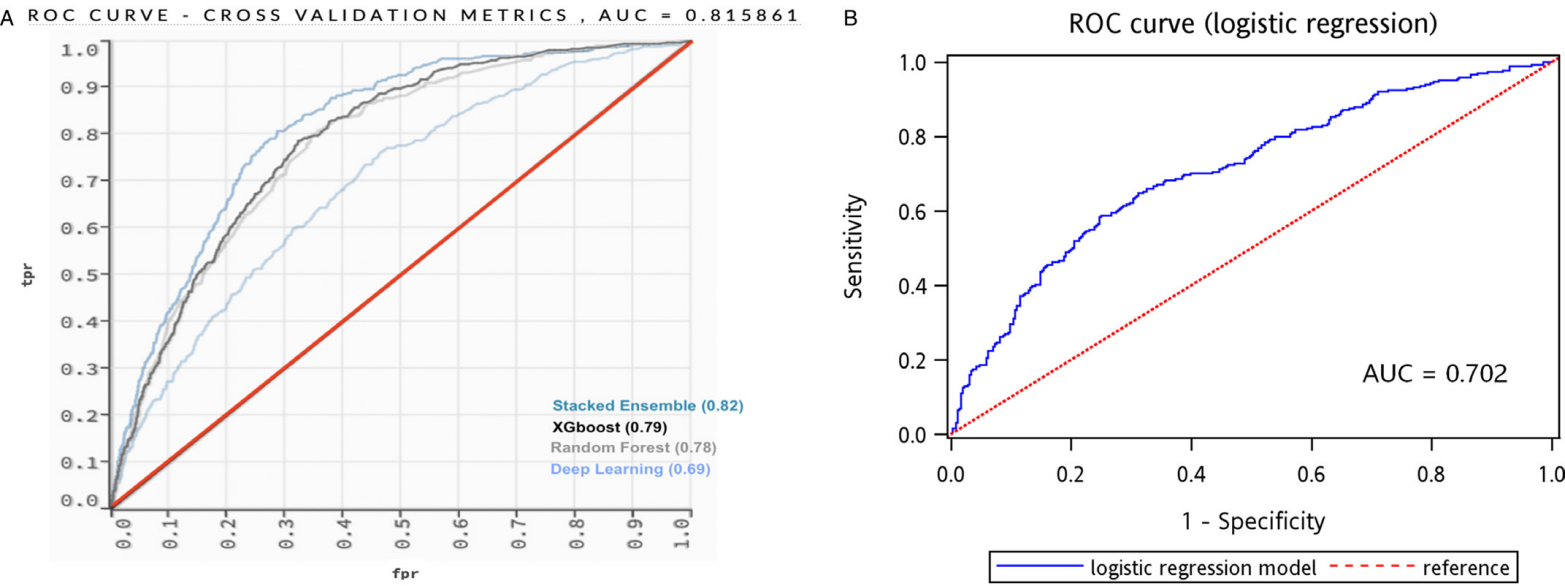
(A) Performance comparison of each AI model. (B) Performance of the logistic regression model. AI, artificial intelligence; AUC, area under the curve; ROC, receiver operating characteristic curve.

Figure 4 is a diagram that displays the true-positive versus false-positive rates and the AUROC that was then calculated. The stacked ensemble model exhibited the highest AUROC (0.82), which was significantly higher than that in the XGBoost model (0.79), random forest model (0.78) and deep-learning model (0.69). Table 2 further summarises the performance of each model in terms of accuracy, sensitivity and specificity. Other than AUROC, the four models demonstrated a similar level of accuracy, which ranged from 0.85 to 0.87. All models demonstrated almost the same sensitivity of 0.45. For specificity, the stacked ensemble model achieved 0.90, followed by the random forest model (0.88), XGBoost model (0.88) and deep-learning model (0.75).

**Table 2:** The comparison of performance in each model

	AUC	Accuracy	Sensitivity	Specificity
Logistic regression	0.64	0.81	0.53	0.70
Deep learning	0.69	0.85	0.45	0.75
Random forest	0.78	0.85	0.46	0.88
XGBoost	0.79	0.85	0.45	0.88
Stacked ensemble	0.82	0.87	0.45	0.90

AUC, area under the curve.

## Discussion

In the current study, we developed an ML approach to predict high-risk ED revisit within 72 hours. Age, systolic blood pressure and heart rate were leading features contributing to high-risk ED revisit, followed by diastolic blood pressure and body temperature. In brief, age and vital signs in the index visit could predict high-risk ED revisit. Our results differ from those of previous reports because the features in the prediction model were cleansed and corrected after the data were reviewed and examined by physicians for each patient, rather than using diagnostic coding or database extraction. In addition, we used an ML approach to overcome the non-linearity and high dimensional features that was difficult to process in the traditional logistic regression. We used the stacked ensemble method, which combined several base learners. As a super learner, the stacked ensemble algorithm increased the prediction performance compared with deep learning, XBG and random forest. The AUROC in this method achieved 0.82. Significantly, our findings revealed that the specificity of ML models greatly surpassed that of traditional logistic regression, making them more effective as predictive tools for users.

Our study indicated that only age and vital signs in the index ED visit could predict high-risk ED revisit, which resulted in some consistent and some controversial findings, compared with previous reports.^7 8 11 12 18^ First, vital signs are important at each ED visit because they reflect the disease condition. In a previous case-crossover study, arrival by ambulance, dyspnoea or chest pain on ED presentation, high triage levels, acute change in levels of consciousness, tachycardia (>90 beats/min) and high fever (>39°C) were associated with high-risk ED revisit.^8^ Vital signs including heart rate and body temperature were important risk factors and features in the regression model and prediction model, respectively. Patients with unstable or abnormal vital signs not only reflected immediate urgency but also a potential high-risk revisit if the patient was discharged from the ED. As for other variables, such as high triage level or vital sign-related symptoms, the association was undoubtedly made. Another retrospective study indicated that male sex, ambulance transport at return visit and longer length of stay were associated with higher risks of admission among ED 72-hour return visits.^11^ Age was adjusted after multivariate regression. However, these factors associated with high-risk ED visit may not reflect the disease condition because the chief concerns, diagnosis and laboratory data were not obtained. Whether the factors of male sex, ambulance transport or longer ED stay were associated with specific diseases remains unknown. In addition, another study included older age, multiple comorbidities and worsening severity index as prognostic factors for poor outcome in high-risk ED revisit. In that study, the overall mortality rate was almost 20%.^12^

Also, our report demonstrated that ML would be a better approach to provide a prediction model than multivariate logistic regression, which mainly focuses on the association between dependent and independent variables. The AUROC in this study was almost the same as that in previous studies that used ML technique, approximately 0.74–0.83 with a different algorithm.^13–15^ However, in some studies with multivariate logistic regression, the concordance (C)-statistic, which is often used to assess the ability of a risk factor to predict outcome, ranged from 0.55 to 0.74.^4^ This finding is not surprising because the cause of high-risk ED revisit was multifactorial. The ML approach was suitable for dealing with high dimensional features, non-linear and complicated features, not simply applied to administrative data.^19 20^ To enhance the performance of the current model, subgroup analysis may be considered, especially considering that certain cohorts, such as patients aged over 75 years old. In our comparison with traditional multivariable logistic regression, we observed notable differences in the variables identified as significant compared with those in the ML model. Table 3 presents the results of the multivariable logistic regression model, which was developed using a stepwise selection process. Several factors were consistently associated with high-risk ED revisits across both the logistic regression and ML models. These included age, sex, systolic blood pressure, the presence or absence of fever, serum levels of white cell count or creatinine, time until return to the ED and issues related to tube malfunction (device issue). However, it was important to highlight that some symptoms such as chest pain, abdominal pain and leg oedema, which might be intuitively assumed as critical, did not emerge as key features in the ML models. Furthermore, certain pre-existing diseases such as chronic obstructive pulmonary disease and malignancy, identified as risk factors for high-risk ED revisits in the logistic regression model, were obviously absent in the ML model. Additionally, our analysis revealed that some features were deemed significant in the ML model but did not demonstrate a similar impact in the logistic regression model. These features included serum levels of electrolytes, the month of the ED visit and liver function tests. Such disparities underscore the distinct analytical perspectives offered by different model approaches. The ML model, with its ability to capture complex interactions and non-linear relationships, may identify subtle patterns not apparent in traditional logistic regression analysis. This difference in model sensitivity and specificity highlighted the need to carefully interpret and understand the implications of each model’s findings, particularly in the context of predicting high-risk ED revisits.

**Table 3:** Logistic regression model with stepwise selection

Variable	aOR	95% CI	P value
Age	1.019	(1.014 to 1.024)	<0.001
Male	1.305	(1.095 to 1.555)	0.003
Systolic blood pressure	0.992	(0.990 to 0.995)	<0.001
Body temperature (fever)	1.496	(1.360 to 1.645)	<0.001
White cell count (x10ˆ9/L)	1.086	(1.061 to 1.111)	<0.001
Serum creatinine (mg/dL)	1.073	(1.024 to 1.124)	0.003
Return to ED			
<24 hours	Ref		
24–48 hours	0.957	(0.787 to 1.164)	0.155
48–72 hours	0.683	(0.540 to 0.864)	0.005
Malignancy	1.426	(1.131 to 1.801)	0.003
COPD	1.891	(1.229 to 2.908)	0.004
Triage level 1 or 2	1.551	(1.234 to 1.948)	0.001
Chest pain	0.576	(0.425 to 0.781)	0.001
Abdominal pain	1.388	(1.140 to 1.689)	0.001
Leg oedema	2.004	(1.211 to 3.316)	0.007
Tube malfunction	0.344	(0.146 to 0.809)	0.014

aOR, adjusted OR; COPD, chronic obstructive pulmonary disease; ED, emergency department.

### Study limitations

This study demonstrated several limitations, First, it was a single-centre study, which may cause selection bias. Although the study duration was 3 years and sample size was sufficient, the patient condition or disease type may be restricted to the local region. In addition, if a patient with a potential high-risk ED revisit chose another hospital after ED discharge, the study may fail to include these cases. Second, because high-risk ED revisit is multifactorial, some features were not collected, which may cause information bias, particularly for qualitative features. One study indicated that the patient being told to ‘return if unwell’ (22.7%) and being seen faster after returning to the ED (12.5%) were associated with ED revisit^21^; however, this information could not be obtained from medical records. Third, the patients in this study were those who had both the index and return ED visit in our hospital. Some patients if they had return visit to other hospitals could not be controlled. Fourth, high-risk ED revisit was also associated with uncertain or missed diagnoses, causing inappropriate dispositions,^22^ but we did not further follow the misdiagnosis rate. Fifth, the study faced a challenge with an unbalanced population, where only 4% were rehospitalised. Consequently, the positive predictive value, a critical metric for users, was merely 0.16, indicating a need for improvement in future studies. Sixth, the first 26 characteristics accounted for only 8% of the model’s performance, as illustrated in figure 3. This suggests that the remaining 126 features contribute at most 12% to the model’s efficacy. Considering this, alternative analytical approaches, such as SHAPE analysis, may be worth exploring. Lastly, this model was not validated in another new cohort. Further external validation would be warranted.

## Conclusion

In this study, we used ML to predict high-risk ED revisit. The stacked ensemble algorithm exhibited better prediction performance compared with random forest, XBG and deep-learning models. The leading features in the prediction model were age, systolic blood pressure and heart rate in the index ED visit. To determine whether this ML model can be externally validated in other clinical settings with the same performance, further evaluation is required.

## Data Availability

Data are available on reasonable request.
