# Supplementary material for: Prediction of high-risk emergency department revisits from a machine-learning algorithm: a proof-of-concept study
Source: BMJ Health Care Inform. 2024 Apr 22;31(1):e100859. doi: 10.1136/bmjhci-2023-100859 (PMC11043771; doi:10.1136/bmjhci-2023-100859)
Supplement: Supplementary data [file bmjhci-2023-100859supp001.pdf]

1	year	pre_rhinorrhea	pre_Seg	post_nausea	post_Cr
2	month	pre_n_bleed	pre_Hb	post_vomiting	post_Na
3	type	pre_abdominal_pain	pre_ALT	post_diarrhea	post_K
4	sex	pre_nausea	pre_Cr	post_constipation	post_CRP
5	Age	pre_vomiting	pre_Na	post_flank_pain	post_PH
6	HTN	pre_diarrhea	pre_K	post_LBP	post_HCO3
7	DM	pre_constipation	pre_CRP	post_dysuria	post_CO2
8	CAD	pre_flank_pain	pre_PH	post_frequency	post_LA
9	CVA	pre_LBP	pre_HCO3	post_urine_retention	post_lab_sugar
10	Cancer	pre_dysuria	pre_CO2	post_chills	Same
11	CKD	pre_frequency	pre_LA	post_coldsweat	Infection
12	COPD	pre_urine_retention	pre_lab_sugar	post_skin	Neurology
13	Dementia	pre_chills	post_GCS	post_soreness	Circulation
14	PSY	pre_coldsweat	post_level	post_edema	Respiratory
15	OP	pre_skin	post_SBP	post_device	GI
16	pre_GCS	pre_soreness	post_DBP	post_A_W	GU
17	pre-level	pre_edema	post_P	post_L_W	Musculoskeletal
18	pre_SBP	pre_device	post_R	post_IV_pain	Others
19	pre_DBP	pre_A_W	post_T	post_Abx	__internal_cv_weights__
20	pre_P	pre_L_W	post_O2	post_EKG	Final
21	pre_R	pre_IV_pain	post_sugar	post_Xray	
22	pre_T	pre_Abx	post_headache	post_CT	
23	pre_O2	pre_EKG	post_dizziness	post_MRI	
24	pre_sugar	pre_Xray	post_vision	post_PES_CFS	
25	pre_headache	pre_CT	post_neck_pain	post_consult	
26	pre_dizziness	pre_MRI	post_chest_pain	post_procedure	
27	pre_vision	pre_PES_CFS	post_numbness	post_out_pain	
28	pre_neck_pain	pre_consult	post_weakness	post_out_abx	
29	pre_chest_pain	pre_procedure	post_dyspnea	post_discharge	
30	pre_numbness	pre_out_pain	post_cough	post_WBC	
31	pre_weakness	pre_out_abx	post_rhinorrhea	post_Seg	
32	pre_dyspnea	pre_discharge	post_n_bleed	post_Hb	
33	pre_cough	pre_WBC	post_abdominal_pain	post_ALT	

Type	Variables
Basic characteristics	Age
	Sex
	Type
Past history	HTN
	DM
	CKD
	CAD
	CVA
Index ED visit	Month
	prelevel
Vital signs	pre_T
	pre_P
	pre_R
chief complaints	pre_abdominal_pain
	pre_chest_pain
	pre_chills
Managements	pre_Abx
	pre_consult
	pre_CT
	pre_EKG
Lab data	pre_Hb
	pre_WBC
	pre_Seg
	pre_ALT
